# Neuralgias

**Published:** 1829

**Authors:** 


30.—*Neuralgias.*—Time was, when a painful affection of the 2d branch of the 5th pair of nerves was almost the only complaint to which the term neuralgia was applied. Matters are very different at present. The books abound in cases of this malady. From a change in the character of diseases, or from new analytical views, we now have neuralgias of all the limbs, of the trunk, of the viscera, and of the organs of sense. The greatest collection of cases of this kind, which we have seen, has been lately published by the indefatigable Professor Warren, in the second volume of the Boston Medical and Surgical Journal. As the Professor has devoted much attention to this interesting subject, we shall enrich our pages with such an extract from his last communication, as will set forth his general therapeutic conclusions.

*General view of the Treatment of this Disease.*—The proper treatment will vary in some respects according to the nerve affected. The different cases which have been stated will show what treatment appeared to me most proper in each of the species; but as I do not expect that those into whose hands these papers may fall will take the trouble to read all I have written, I shall condense the most important parts of the treatment in a few words.

The first point to be settled is the seat of the disease, or the precise nerve affected. This can usually be done by the sensation of the patient, and a proper knowledge of the course of the nerves. Next, we must investigate the condition of the digestive organs, and administer proper remedies, if required; since it has appeared that the disease sometimes depends on, and is often aggravated by, a derangement in that part of the animal economy. Then, leeches are to be applied as nearly as possible to the affected part, and repeated two or three times a week; or if the patient is of a weak constitution, or in an exhausted state from disease, the best application would be blisters, employed in a series, for two or three weeks, and of as large size as the part affected will admit. When the patient is full of blood, venesection is very proper; but this is rarely the case. The disease not yielding to these remedies, I should put the patient on a course of Carbonate or Sulphate of Iron. The former, if the nerves of the head were the seat of the disease, in the dose of from one to two drachms, three times a day; the latter, if the trunk or extremities were affected, to the amount of three grains, four times in twenty-four hours, at the same time using the hot douche. After a proper use of such remedies for the space of six weeks, I should propose to excise a portion of the disordered nerve, whenever its situation rendered the operation practicable. Should this not be the fact, then may be employed the other remedies mentioned, especially Conium, Stramonium, and Opium internally, and the cautery, actual or potential, externally.

The following remarks on the nature and treatment of this complaint, occur to me as of sufficient importance to be presented in a distinct form.

1. Neuralgia is a disease of the nerves. The muscles are disordered in a secondary way only.

2. This affection is not confined to the head. All the principal nerves of the body are liable to be affected with it, but superficial nerves more than others.

3. After being subdued on the first attack, it is apt to return on the application of exciting causes, and to become one of the most intractable of complaints.

4. There is no remedy which can be considered a specific cure for it. There is no one remedy that is often successful. It is to be combatted by various agents judiciously adapted to the peculiarities of the case.

5. Of consequence, the Carbonate of Iron is not entitled to the praises which have been bestowed on it; nor is Hemlock, Stramonium, or Belladonna.

6. A surgical operation for excision of the affected nerve is frequently successful in the early part of the disease, and often fails in a protracted case, even when it gives temporary relief.

7. This operation, when successful, is not always followed by immediate disappearance of the paroxysms.

8. The affusion of hot water is a valuable remedy, when the disease is seated in an extremity.

9. The physical and intellectual constitution of the patient is to be studied carefully at first, and kept in view during the whole course of the disease.

10. The patient is never to be abandoned to his sufferings. When the more approved remedies have been tried, the most extraordinary practice is justifiable.

